# Berberine-entrapped albumin nanoparticles ameliorate chemically induced liver injury by restoring oxidative balance and autophagic-apoptotic crosstalk

**DOI:** 10.1038/s41598-026-43119-1

**Published:** 2026-03-27

**Authors:** Heba Zaied, Mohamed I. Ashmawy, Ahmed E. Abdel Karim, Doaa A. Ghareeb, Abeer El Wakil

**Affiliations:** 1 Department of Biological and Geological Sciences, Faculty of Education, Alexandria University, Alexandria, 21526 Egypt; 2 Department of Pharmaceutical Chemistry, Faculty of Pharmacy, Alexandria University, Alexandria, Egypt; 3 Department of Zoology, Faculty of Science, Alexandria University, Alexandria, Egypt; 4 Bio-screening and Preclinical Trial Lab, Biochemistry Department, Faculty of Science, Alexandria University, Alexandria, Egypt

**Keywords:** Natural product, Molecular docking, Sigma1-receptor, DEN/CCl_4_, Biochemistry, Cancer, Cell biology, Diseases, Drug discovery

## Abstract

This study investigated the therapeutic potential of berberine-entrapped bovine serum albumin nanoparticles (BRB-BSA NPs) in alleviating chemically induced liver injury in rats. Molecular docking was first performed to examine BRB interactions with phosphoinositide 3-kinase (PI3K), a key regulator of cellular survival and autophagy pathways. Hepatotoxicity was induced using diethylnitrosamine (DEN) and carbon tetrachloride (CCl₄), resulting in significantly elevated serum uric acid levels (1.35 ± 0.1 mg/dL), oxidative imbalance, disrupted autophagic signaling, and histological liver damage. Post-injury treatment with BRB-BSA NPs significantly reduced serum uric acid (0.20 ± 0.07 mg/dL, *p* < 0.05 vs. DEN/CCl_4_), surpassing the prophylactic regimen and restoring levels comparable to healthy controls. Oxidative status improved, with increased superoxide dismutase (SOD) activity and reduced nitric oxide (NO) and xanthine oxidase (XO) levels. Autophagic signaling was normalized through downregulation of PI3K, mTOR, and p62, alongside upregulation of LC3, indicating restoration of autophagic flux. Apoptotic balance shifted toward pro-apoptotic signaling, with elevated Bax and reduced Bcl-2 expression, supporting the therapeutic potential that BRB-BSA NPs may exert. Histological assessment confirmed near-complete hepatic architecture recovery in the treatment group, while the prophylactic group exhibited partial protection. Collectively, these findings highlight the potent therapeutic role of BRB-BSA NPs in reversing DEN/CCl_4−_ induced hepatic damage by restoring metabolic, oxidative, autophagic, and apoptotic homeostasis, underscoring their promise as a nanoformulated hepatoprotective intervention.

## Introduction

The liver is the body’s primary metabolizing organ, essential for maintaining homeostasis across various systems. Its major functions include protein synthesis, fat and carbohydrate metabolism and storage, detoxification of drugs and toxins, bilirubin excretion, and hormone regulation^1^. Hepatocellular carcinoma (HCC) is a leading cause of cancer-related death in the world, making it a serious public health problem. It is a complex disease related to various potential risk factors, such as inflammation, fibrosis, cirrhosis, and DNA damage^2^. Diethylnitrosamine (DEN) is a potent hepatocarcinogen widely used to induce progressive liver injury in experimental models. However, depending on dose and duration, DEN may produce either chronic hepatic damage or full HCC; in shorter protocols, it induces pre-carcinogenic injury rather than fully developed HCC. Combining DEN with carbon tetrachloride (CCl₄) further elevates endotoxin levels and aggravates hepatic injury, mimicking early pathological features of the clinical microenvironment associated with HCC development without necessarily inducing overt tumor formation^3^.

Conventional cancer treatments face challenges like drug resistance and cancer relapse^4^. Thus, developing novel hepatoprotective or anticancer agents remains critical. Plant extracts, as potential therapeutic agents, show promise in alleviating early hepatic injury and serving as adjuvant therapies. Berberine (BRB), an isoquinoline alkaloid derived from *Berberis* plants, is known for its antimicrobial, antiprotozoal, and anticancer properties^5^. However, oral administration of BRB poses challenges due to its rapid metabolism and poor absorption. The liver is the primary organ of BRB distribution, with concentrations 70 times higher than in plasma^6^. Recent advances have focused on nanostructure carriers to enhance BRB’s aqueous solubility and intracellular concentration^7^. Bovine serum albumin nanoparticles (BSA NPs), a non-toxic, biodegradable, and low-cost material, are widely used as drug carriers due to their functional surface groups (thiol, amine, and carboxylic), enabling drug conjugation^8^. By combining the bioactive potential of BRB with the drug delivery capabilities of BSA NPs, BRB-entrapped albumin nanoparticles (BRB-BSA NPs) represent a promising therapeutic strategy for addressing DEN/CCl_4_-induced hepatic injury and potentially mitigating early pathological events that precede malignant transformation. The nanoparticle formulation utilizes a 10:1 BSA-to-BRB ratio, as previously detailed and characterized in our earlier publication^9^.

In HCC biology, the phosphoinositide 3-kinase (PI3K)/AKT/mTOR pathway is a critical driver of tumor proliferation and progression. Drugs targeting this pathway can inhibit tumor growth by promoting autophagy and apoptosis^10,11^. BRB has been shown to inhibit cancer cell growth and metastasis in laryngeal carcinoma by blocking the PI3K/AKT/mTOR signaling pathway^12^. Additionally, BRB induces apoptosis in HCC by modulating the BAX/BCL2 regulatory pathway and increasing LC3 expression, an autophagic biomarker^13^.

We hypothesized that BRB-BSA NPs could exert therapeutic effects against early stage chemically induced hepatic injury relevant to the initial phases of HCC, due to their diverse biological activities. To support this hypothesis, molecular modeling was first performed to examine the potential interactions of BRB compared with copanlisib at the active site of PI3K. Copanlisib, a potent and selective pan-class I PI3K inhibitor, demonstrates strong activity against the PI3K-α and PI3K-δ isoforms, key mediators of survival signaling^14,15^. Its well-characterized crystallographic complex with PI3K (PDB ID: 5G2N) makes it an ideal reference compound for docking validation, ensuring accuracy in binding pose prediction. Following the molecular modeling, we investigated the in vivo potential of BRB–BSA NPs in mitigating hepatic injury induced by DEN/CCl₄ in male albino rats, using both protection and treatment protocols. Multiple parameters were assessed, including serum uric acid, oxidative and antioxidant markers, autophagy and apoptotic indicators, and histopathological alterations in liver tissue.

## Materials and methods

### Chemicals

BRB, in the form of berberine chloride hydrate (CₒH₁₈ClNO₄·xH₂O), was obtained from Alfa Aesar (CAS: 141433-60-5, Thermo Fisher Scientific, Germany). All other reagents used were of analytical grade.

### Molecular docking

The crystallographic structure of the PI3K–Copanlisib complex (PDB ID: 5G2N) was retrieved from the Protein Data Bank. All molecular docking simulations were performed using the Molecular Operating Environment (MOE) software, version 2020.09^16^.

For ligand preparation, copanlisib was processed by adding hydrogen atoms, calculating partial charges, and performing energy minimization using the Amber10:EHT force field, with a convergence criterion of 0.1 kcal/mol·Å and root mean square deviation (RMSD) restraints.

For protein preparation, redundant chains were removed to simplify the model, and structural optimization was performed using the MOE QuickPrep protocol, which included partial charge assignment, 3D protonation, and energy minimization to ensure structural stability.

The MOE Dock module was applied to predict optimal binding modes and affinity scores for Copanlisib through an induced-fit refinement approach. To validate the docking methodology, the co-crystallized ligand PIKiN3 was re-docked into the PI3K active site, yielding an RMSD of 0.7574 Å, confirming the reliability of the docking protocol. The triangle matcher placement method combined with the London dG scoring function was used as the primary scoring scheme, followed by induced-fit refinement and rescoring using the affinity dG function. Docked poses were ranked according to binding affinity, and the top-scoring conformations showing optimal interactions with the active site residues were selected for further analysis.

### Experimental animals and study design set-up

Thirty healthy male Wistar albino rats, four weeks old weighing approximately 100 ± 10 g, were utilized in this study. The animals were sourced from the Faculty of Science, Alexandria University, Egypt, and housed in the institution’s animal facility under standard laboratory conditions. They were maintained in clean polypropylene cages with controlled temperature (24 ± 3 °C), relative humidity (40–60%), and a 12-hour light/dark cycle. Rats had free access to standard rodent chow and tap water. Prior to the start of the experiment, they were allowed to acclimate to the laboratory environment for two weeks.

Following one week of acclimatization, the rats were randomly assigned into five groups (*n* = 6 per group) as follows: (1) Control Group received no treatment throughout the 12-week experimental duration. (2) Vehicle/NP Group was administered 1 mL of blank BSA NPs orally at a dose of 100 mg BSA/kg/day for four weeks^9,17,18^. After this initial phase, animals received an equivalent volume of saline for the remaining eight weeks to match the duration of exposure across all groups. (3) Induction Group received DEN at 70 mg/kg intraperitoneally in 1 mL/kg^19^, together with 375 µL/kg body weight of CCl₄ diluted in olive oil^20^, administered once weekly for eight weeks. No further treatment was given during the remaining four weeks. (4) Protection Group was administered 1 mL of BRB-BSA NPs orally at a BRB dose of 10 mg/kg/day (corresponding to approximately 100 mg BSA/kg/day based on the 10:1 BSA-to-BRB formulation ) for four weeks prior to the initiation of DEN/CCl₄ injections. (5) Treatment Group received the same DEN/CCl₄ induction protocol as the Induction Group, followed by oral administration of 1 mL BRB-BSA NPs at a BRB dose of 10 mg/kg/day (~ 100 mg BSA/kg/day) for four weeks. BRB-BSA NPs were freshly prepared in distilled water before administration.

At the end of the 12-week experimental period, all animals were fasted for 12 h, anesthetized with 5% isoflurane, and euthanized by cervical dislocation for subsequent sample collection and analysis. Euthanasia was performed only after confirming that the animals were fully unconscious under deep isoflurane anesthesia to ensure the absence of pain perception. At the time of euthanasia, the animals weighed on average 350 ± 25 g.

### Experimental ethics

All experimental procedures were conducted in accordance with the guidelines outlined in the Guide for the Care and Use of Laboratory Animals and were approved by the Institutional Animal Care and Use Committee (IACUC) of the Scientific Research and Technological Applications City (Approval No. 31-1Z-1120). The study was also carried out in full compliance with the ARRIVE guidelines.

### Sampling and liver tissue collection

Following sacrifice, blood was collected via cardiac puncture into test tubes. The samples were centrifuged at 5000 rpm for 5 min at room temperature to separate the serum. Isolated sera were stored at − 20 °C until biochemical analyses were conducted. A portion of the liver was stored at − 80 °C for biochemical evaluation. Another portion was minced and homogenized (10% w/v) in ice-cold phosphate-buffered saline (PBS, 0.25 M, pH 7.4) using a Potter–Elvehjem homogenizer. The homogenates were then centrifuged at 15,000 rpm for 15 min at 4 °C to remove cellular debris, and the resulting supernatants were collected and stored at − 80 °C for subsequent analyses. Additional liver samples were immediately fixed in 10% formalin for histological examination.

### Biochemical analyses

#### Uric acid levels

Serum uric acid levels were determined using the enzymatic colorimetric method^21^. The assay wasperformed according to the manufacturer’s protocol, and absorbance was measured at 515 nm using aspectrophotometer. Uric acid concentrations were calculated based on a standard curve and expressed inmg/dL.

#### Oxidant and antioxidant parameters

Nitric oxide (NO) concentration was measured using the method of Montgomery and Dymock^22^, which relies on the spectrophotometric detection of the chromophore formed during the reaction. Absorbance was recorded at 540 nm and NO levels were quantified using a standard curve. Results are expressed as nmol/mg protein.

Xanthine oxidase (XO) activity, a sensitive marker of jaundice in acute liver injury, was assessed according to the method of Litwack et al.,^23^. Absorbance was measured spectrophotometrically at 660 nm. Enzymatic activity was calculated and expressed as mU/mg protein.

Superoxide dismutase (SOD) activity was determined based on the method described by Marklund and Marklund^24^. The assay involves spectrophotometric measurement of color intensity at 420 nm, with enzyme activity calculated accordingly.

#### Quantification of autophagy and apoptosis markers in liver tissue using enzyme-linked immunosorbent assay (ELISA)

To assess the autophagy signaling pathway, the concentrations of key regulatory proteins, including phosphatidylinositol 3-kinase (PI3K; Cat. #MBS702819), p62/sequestosome 1 (p62/SQSTM1; Cat. #MBS2122235), and mammalian target of rapamycin (mTOR; Cat. #MBS744326), were quantified in liver tissue homogenates using commercially available ELISA kits (MyBioSource Inc., San Diego, CA, USA), in accordance with the manufacturers’ instructions. In addition, apoptosis-related changes were assessed by quantifying the protein levels of the anti-apoptotic marker B-cell lymphoma 2 (Bcl-2; Cat. #CSB-E08854r) and the pro-apoptotic marker Bcl-2-associated X protein (Bax; Cat. #CSB-EL002573RA) in tissue homogenates using commercially available ELISA kits (Cusabio, Houston, TX, USA), according to the manufacturers’instructions.

#### Evaluation of autophagy biomarker in liver tissue by Western blotting

The expression of the autophagy-related protein microtubule-associated protein 1 light chain 3 (LC3) was analyzed by Western blotting. Autophagy-related changes were assessed by quantifying total LC3B protein expression. Western blot analysis was performed on three randomly selected biological replicates per group, selected prior to blotting to avoid any bias or data-driven sample selection. This subset size reflects technical feasibility constraints, including limited remaining sample volume after biochemical assays and restricted membrane capacity. Liver tissue lysates were prepared and subjected to SDS-PAGE, followed by transfer to polyvinylidene difluoride (PVDF) membranes. Membranes were incubated overnight at 4 °C with a primary antibody against LC3B (Catalog #2775S, Cell Signaling Technology, USA). The following day, they were probed with appropriate secondary antibody at room temperature. *β*-actin (AC-15) (Catalog #NB600-501, Novus Biologicals, USA) served as a loading control. Densitometric analysis of protein bands was performed using ImageJ software.

### Histopathology

Liver sections were histologically evaluated using hematoxylin and eosin (H&E) staining to assess the protective or therapeutic effects of BRB-BSA NPs on hepatic tissue architecture, compared to the damage induced by DEN/CCl₄. Liver samples were fixed in 10% neutral-buffered formalin immediately after collection, processed using an automated tissue processor, and embedded in paraffin. Tissue blocks were sectioned at a thickness of 4–6 μm using a microtome. The sections were then stained with hematoxylin and counterstained with eosin for microscopic examination. Histological images were captured using an Olympus XC30 microscope equipped with a digital Olympus UC30 camera. Representative images from each experimental group were taken at 400× magnification with a calibrated scale bar of 100 μm.

### Statistical procedures

Statistical analyzes were assessed using one-way analysis of variance (ANOVA) with SPSS version 22 (SPSS, IBM, USA). Post hoc comparisons between groups were conducted using Duncan’s Multiple Range Test (DMRT;^25^, which is appropriate for exploratory mechanistic animal studies due to its higher sensitivity in small sample sizes. Data are presented as mean ± standard error (SE), and *p* ˂ 0.05 was considered statistically significant.

### Uric acid levels

Serum uric acid levels were determined using the enzymatic colorimetric method^21^. The assay was performed according to the manufacturer’s protocol, and absorbance was measured at 515 nm using a spectrophotometer. Uric acid concentrations were calculated based on a standard curve and expressed in mg/dL.

## Results

### Molecular docking of BRB into the active site of PI3K

To explore the potential mechanism underlying the hepatoprotective effects of BRB, molecular docking studies were conducted targeting PI3K, a protein frequently overexpressed in HCC. Docking simulations were performed using the MOE 2020.09 software with the crystallographic structure of the PI3K catalytic domain bound to copanlisib (PDB ID: 5G2N)^16^.

To validate the docking protocol, the co-crystallized ligand copanlisib was re-docked into the PI3K active site, yielding an RMSD of 0.7574, confirming the reliability of the method. BRB demonstrated a binding free energy of − 8.575 kcal/mol, which was comparable to copanlisib’s − 9.846 kcal/mol.

**Fig. 1:**
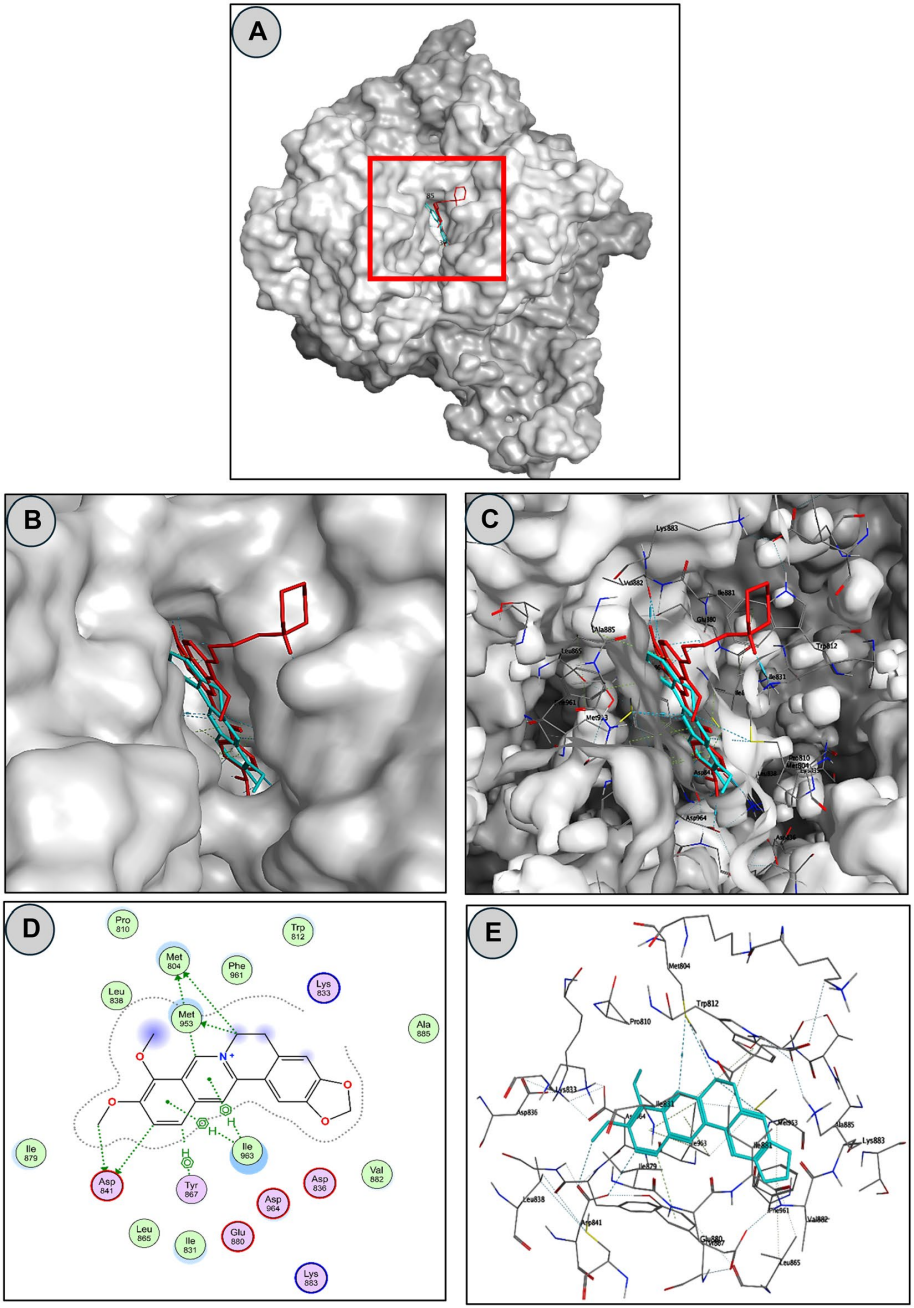
Binding mode of BRB with PI3K (PDB ID: 5G2N). (**A**) Cartoon representation of the PI3K molecular surface showing BRB positioned within the binding pocket. (**B**) Enlarged inset from panel A highlighting the PI3K active site. (**C**) Transparent surface representation of PI3K illustrating key amino acid interactions within the binding pocket. (**D**) Two-dimensional interaction diagram of BRB with PI3K. (**E**) Three-dimensional representation of BRB–PI3K interactions.

Docking analysis revealed that BRB was stably accommodated within the PI3K binding site through a combination of polar and hydrophobic interactions (Fig. 1A–C). Key contacts included hydrogen bonds between the isoquinoline ring of BRB and Met804 and Met953, as well as an interaction between a methoxy group and Asp841. Additionally, the isoquinoline moiety formed arene–hydrogen interactions with Tyr867, Ile963, Ile411, and Ile347 (Fig. 1D and E). These findings, supported by the interaction profile and binding energy values summarized in Table 1, suggest that BRB exhibits a binding affinity to PI3K comparable to that of copanlisib.

**Table 1:** Binding energies and interactions of copanlisib and BRB with PI3K (PDB ID: 5G2N) active site.

Compound	Docking scores (Kcal/mol)	Hydrogen bond	Other interactions
		Residues involved (distance Å)	Residues involved (distance Å)
Copanlisib	−9.846	Lys802 (2.90) Lys833 (2.86) Asp841 (2.95) Glu880 (2.97) Val882 (2.87) Asp964 (3.02)	
BRB	−8.575	Met804 (3.97) Asp841 (3.45) Met953 (3.77)	Ile963 (4.15)

### Therapeutic potential of BRB-BSA NPs in regulating serum uric acid in DEN/CCl_4_ -induced liver injury

**Fig. 2:**
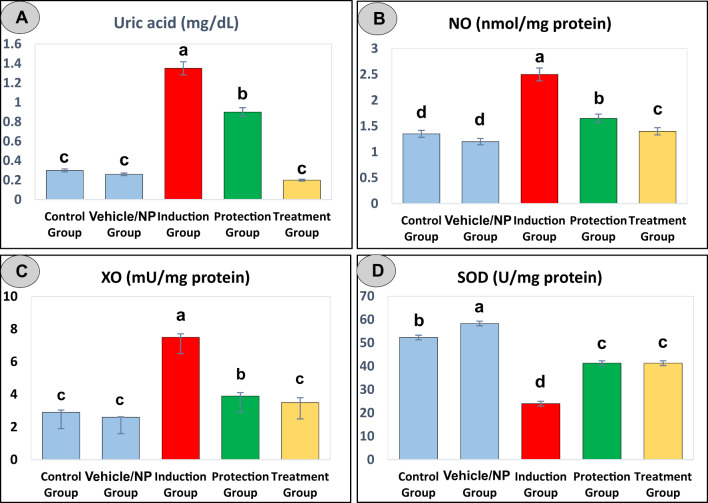
Effects of BRB-BSA nanoparticles on serum uric acid and hepatic oxidative stress/antioxidant markers in rats. Levels of (**A**) serum uric acid, (**B**) hepatic nitric oxide (NO), (**C**) xanthine oxidase (XO), and (**D**) superoxide dismutase (SOD) were measured across experimental groups. BRB-BSA NPs treatment modulated these parameters, reflecting their potential role in reducing oxidative stress and enhancing antioxidant defense. Data are presented as mean ± SE (*n* = 6). Columns sharing the same letter within each panel are not significantly different (*p* < 0.05), as determined by one-way ANOVA followed by Duncan’s multiple range post hoc test.

A significant increase in serum uric acid levels was observed in the DEN/CCl₄-exposed group (1.35 ± 0.1 mg/dL) compared to both the control (0.30 ± 0.11 mg/dL) and vehicle/NP (0.26 ± 0.06 mg/dL) groups (*p* < 0.05), reflecting pronounced hepatic dysfunction and oxidative stress typically associated with chemically induced liver injury (Table 2; Fig. 2A). Elevated uric acid is a recognized biomarker of altered purine metabolism and impaired hepatic clearance under toxic conditions. Remarkably, therapeutic administration of BRB-BSA NPs led to a substantial and statistically significant reduction in uric acid levels (0.20 ± 0.07 mg/dL, *p* < 0.05 vs. DEN/CCl₄ group), with a more pronounced effect than that observed in the group receiving BRB-BSA NPs as a protective intervention (0.9 ± 0.05 mg/dL). The uric acid concentrations in the treatment group nearly returned to baseline values observed in the control and vehicle/NP groups, indicating robust hepatoprotective activity. These findings suggest that BRB-BSA NPs not only ameliorate oxidative damage but also help restore purine metabolism, likely through their antioxidant mechanism of action.

**Table 2:** Statistical analysis of hepatic oxidative stress markers across experimental rat groups. Data was expressed as Mean ± SE. Post hoc comparisons between groups were conducted using Duncan’s Multiple Range Test (DMRT). Means that do not share the same letter are significantly different (*n* = 6).

Experimental groups	Parameters			
	Uric acid (mg/dL)	NO (nmol/mg protein)	XO (mU/mg protein)	SOD (U/mg protein)
Control group	0.30 ± 0 .11^c^	1.35 ± 0.1^d^	2.9 ± 0.14^c^	52.33 ± 3.2^b^
Vehicle/NP group	0.26 ± 0.06^c^	1.2 ± 0.1^d^	2.6 ± 0.03^c^	58.33 ± 2.03^a^
Induction group	1.35 ± 0.1^a^	2.5 ± 0.04^a^	7.5 ± 0.21^a^	24 ± 1.15^d^
Protection group	0.9 ± 0.05^b^	1.65 ± 0.1^b^	3.9 ± 0.21^b^	41.33 ± 2.4^c^
Treatment group	0.20 ± 0.07^c^	1.4 ± 0.04^c^	3.5 ± 0.3^c^	41.33 ± 1.8^c^

### Modulatory impact of BRB-BSA NPs on DEN/CCl_4_-induced oxidative stress and antioxidant enzymes

Compared to both the control and vehicle/NP groups, adult male rats administered DEN and CCl₄ exhibited a significant increase in hepatic levels of NO and XO, along with a marked decrease in SOD activity (*p* < 0.05 for all), indicating enhanced oxidative stress and impaired antioxidant defense mechanisms (Table 2; Fig. 2B–D). These alterations reflect the pathological oxidative environment induced by chemical hepatotoxins, which promote free radical generation and lipid peroxidation as demonstrated in our previous study^18^. Notably, treatment with BRB-BSA NPs significantly reversed these effects. BRB-BSA NPs-treated rats displayed a substantial elevation in SOD activity, suggesting improved antioxidant capacity, alongside a significant reduction in NO and XO levels (*p* < 0.05 vs. DEN/CCl₄ group), indicating attenuation of oxidative and nitrosative stress. These findings highlight the potent antioxidative and hepatoprotective properties of BRB-BSA NPs in mitigating DEN/CCl₄-induced liver injury.

### Evaluation of autophagy markers in hepatic tissues across experimental groups

**Fig. 3:**
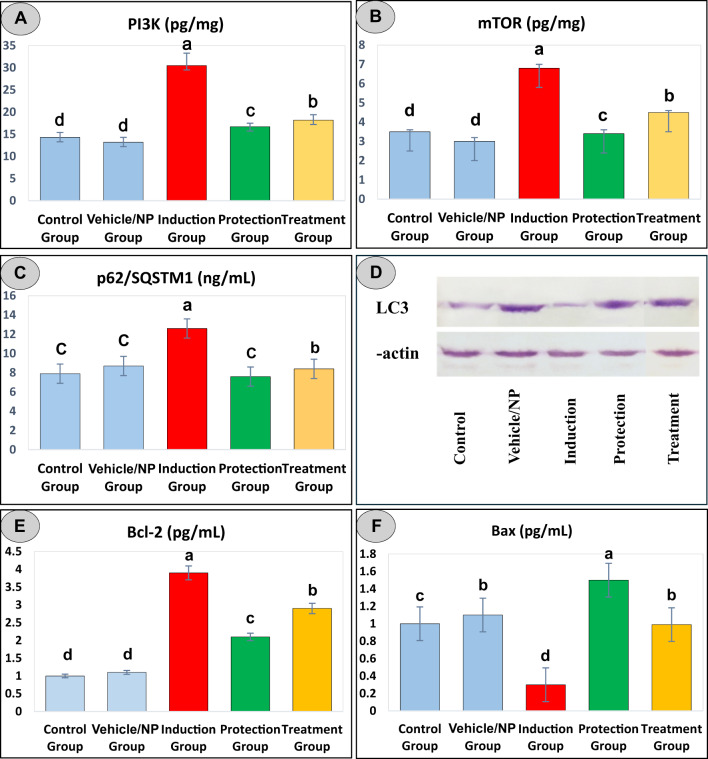
Evaluation of autophagy and apoptosis markers in hepatic tissues across experimental groups. Quantitative analysis of key autophagy-related proteins in liver homogenates, including (**A**) phosphoinositide 3-kinase (PI3K), (**B**) mammalian target of rapamycin (mTOR), (**C**) p62/sequestosome 1 (p62/SQSTM1), and (**D**) microtubule-associated protein light chain 3 (LC3). These markers reflect the status of autophagic signaling and flux in response to experimental interventions. (**E**) anti-apoptotic marker Bcl-2 and (**F**) pro-apoptotic marker Bax. Data are presented as mean ± SE (*n* = 6 for all measures, except LC3, for which *n* = 3). Columns sharing the same letter within each panel are not significantly different (*p* < 0.05), as determined by one-way ANOVA followed by Duncan’s multiple range post hoc test.

Rats exposed to DEN/CCl₄ exhibited a significant upregulation of PI3K and mTOR in hepatic tissues, with levels reaching 30.5 ± 2.8 pg/mg and 6.8 ± 0.2 pg/mg, respectively (Table 3; Fig. 3A and B). These values were markedly higher than those observed in the control group (14.3 ± 1.1 pg/mg and 3.5 ± 0.1 pg/mg) and the vehicle/NP group (13.2 ± 1.1 pg/mg and 3 ± 0.2 pg/mg) (*p* < 0.05), indicating significant suppression of autophagy initiation. Additionally, the expression of p62 (also known as sequestosome 1), a selective autophagy adaptor that accumulates when autophagic degradation is impaired, was significantly elevated in the DEN/CCl₄ group (12.6 ± 0.19 ng/mL) (Table 3; Fig. 3C), further supporting the presence of disrupted autophagic flux and insufficient clearance of cellular waste.

**Table 3:** Statistical analysis of autophagy markers across experimental rat groups.

Experimental groups	Parameters		
	PI3K (pg/mg)	mTOR (pg/mg)	p62/SQSTM1 (ng/mL)
Control group	14.3 ± 1.1^d^	3.5 ± 0.1^d^	7.9 ± 2.4^c^
Vehicle/NP group	13.2 ± 1.1^d^	3 ± 0.2^d^	8.7 ± 0.1^c^
Induction group	30.5 ± 2.8^a^	6.8 ± 0.2^a^	12.6 ± 0.19^a^
Protection group	16.7 ± 0.8^c^	3.4 ± 0.2^c^	7.6 ± 1.2^c^
Treatment group	18.2 ± 1.2^b^	4.5 ± 0.1^b^	8.4 ± 1.8^b^

Data was expressed as Mean ± SE. Post hoc comparisons between groups were conducted using Duncan’s Multiple Range Test (DMRT). Means that do not share the same letter are significantly different (*n* = 6).

In contrast, therapeutic administration of BRB-BSA NPs led to a notable and statistically significant reduction in the levels of PI3K (18.2 ± 1.2 pg/mg), mTOR (4.5 ± 0.1 pg/mg), and p62 (8.4 ± 1.8 ng/mL) compared to the DEN/CCl₄ group (*p* < 0.05), indicating partial restoration of autophagic signaling. Interestingly, the protective administration of BRB-BSA NPs produced an even more pronounced effect, with PI3K (16.7 ± 0.8 pg/mg), mTOR (3.4 ± 0.2 pg/mg), and p62 (7.6 ± 1.2 ng/mL) levels closely approximating those of the control and vehicle/NP groups. These findings suggest that prophylactic treatment may offer superior efficacy in preserving autophagic homeostasis and preventing hepatocellular damage compared to post-injury therapeutic intervention.

Furthermore, the expression of total LC3, a key protein associated with autophagy-related processes, was significantly increased in the BRB-BSA NPs-treated group, exceeding the levels observed in the protective group (Fig. 3D). In contrast, total LC3 expression was markedly reduced in the DEN/CCl₄ group, indicating impaired autophagic flux associated with hepatic injury. Densitometric analysis confirmed this pattern, where the induction group demonstrated the lowest LC3/β-actin ratio, reflecting pronounced downregulation of autophagy-associated protein expression (Table 4). In the treated group, however, the LC3/β-actin ratio was restored to levels comparable to, or even surpassing, those of the control and vehicle/NP groups, demonstrating a robust reactivation of autophagy-related signaling. Normalization of LC3B intensity to β-actin, a consistently expressed housekeeping protein, corrected for loading variability and indicated that the observed differences reflect real biological changes. Collectively, these findings reinforce that BRB-BSA NPs effectively reverse DEN/CCL_4_-induced autophagy inhibition, highlighting their potential to restore cellular homeostasis and mitigate liver damage through modulation of the autophagy pathway.

**Table 4:** Densitometric quantification of total LC3 and β-actin protein bands and the LC3/β-actin ratio in hepatic tissues across experimental rat groups.

Experimental groups	Parameters		
	LC3	β-actin	LC3/β-actin ratio
Control group	7.91 ± 0.11^b^	11.22 ± 0.89^a^	0.71 ± 0.04^b^
Vehicle/NP group	15.75 ± 0.25^d^	12.16 ± 0.91^a^	1.29 ± 0.08^c^
Induction group	5.89 ± 0.27^a^	11.31 ± 0.68^a^	0.52 ± 0.06^a^
Protection group	13.66 ± 0.09^c^	9.32 ± 0.18^a^	1.47 ± 0.04^d^
Treatment group	15.83 ± 0.09^d^	11.32 ± 0.94^a^	1.39 ± 0.06^cd^

Data was expressed as Mean ± SE. Post hoc comparisons between groups were conducted using Duncan’s Multiple Range Test (DMRT). Means that do not share the same letter are significantly different (*n* = 3).

### Modulation of apoptotic markers in response to BRB-BSA NPs treatment

Apoptosis, or programmed cell death, is a tightly regulated process involving pro- and anti-apoptotic proteins. In the present study, DEN/CCl₄-induced hepatic damage in rats was associated with a significant dysregulation of apoptotic markers, as evidenced by an upregulation of the anti-apoptotic protein Bcl-2 and a corresponding downregulation of the pro-apoptotic protein Bax (Fig. 3E and F). This imbalance suggests a suppression of apoptosis, contributing to hepatotumorigenesis. Conversely, treatment with BRB-BSA NPs effectively reversed this pattern, leading to decreased Bcl-2 expression and increased Bax levels. These findings indicate a reactivation of the apoptotic pathway in treated groups, suggesting that BRB-BSA NPs may restore apoptotic balance and counteract tumor progression in hepatic tissues.

### Histopathological changes in the hepatic tissue

**Fig. 4:**
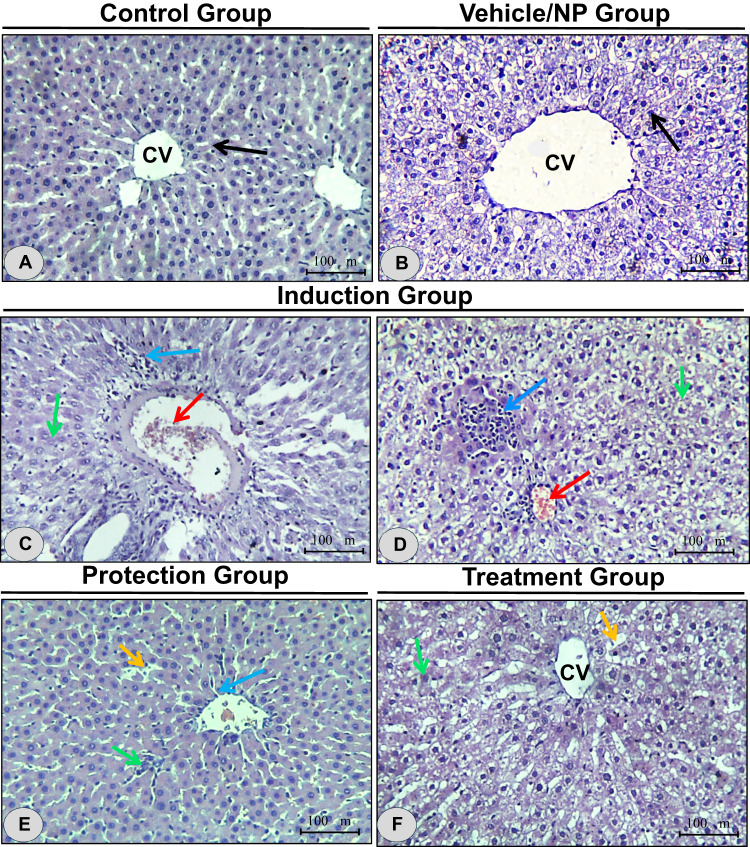
Histopathological evaluation of hepatic tissues across experimental groups. Representative liver sections stained with H&E illustrating histological changes in response to treatments. (**A**, **B**) Control and vehicle/NP groups display normal hepatic architecture with well-organized hepatocyte plates radiating from the central vein (CV). Hepatic strands are indicated by black arrows. (**C**, **D**) DEN/CCl_4−_ treated rats exhibit severe hepatic damage characterized by centrilobular congestion, hemorrhagic foci (), nuclear pyknosis (), and inflammatory cell infiltration (). (**E**) Prophylactic administration of BRB-BSA NPs prior to DEN/CCl_4_ exposure results in partial preservation of liver structure, though protective effects remain limited. Notable histopathological alterations, including sinusoidal dilation (), pyknotic nuclei (), and inflammatory cell infiltration () are still evident. (**F**) Therapeutic administration of BRB-BSA NPs post-injury markedly improves hepatic architecture, with reduced degeneration and inflammation, although mild sinusoidal dilation () and residual pyknotic nuclei () persist. These findings suggest greater efficacy of BRB-BSA NPs in reversing established liver injury compared to prevention. Magnification 400x, scale bar = 100 μm.

The liver histology of both the control and vehicle/NP groups appeared normal and well-preserved (Fig. 4A and B), displaying a typical architecture with hepatocytes arranged in orderly plates radiating from the central vein. In contrast, hepatic tissues from rats administered DEN/CCl₄ exhibited marked histopathological disruptions (Fig. 4C and D). These included focal infiltration of inflammatory cells, nuclear pyknosis, and widespread hepatic degeneration, notably centrilobular congestion and hemorrhagic foci.

In animals receiving BRB-BSA NPs prophylactically prior to DEN/CCl₄ exposure, histopathological alterations were still evident, indicating that structural changes at the tissue level were not entirely prevented within the time frame of the model (Fig. 4E). However, these qualitative features were directionally consistent with improvements in oxidative stress and biochemical parameters in this group. In comparison, therapeutic intervention with BRB-BSA NPs following DEN/CCl₄-induced injury appeared to show a clearer qualitative recovery of hepatic architecture (Fig. 4F); however, this observation is descriptive and should be interpreted in light of the quantitative biochemical evidence rather than viewed as a standalone ranked outcome. Minor residual abnormalities, such as sinusoidal dilation and isolated pyknotic nuclei, were still observed.

Because histology in this study was descriptive and was not designed for semi-quantitative scoring, these observations are intended to visually corroborate biochemical endpoints rather than to independently determine comparative efficacy. Additionally, it is plausible that the limited prophylactic impact reflects the non-overlapping duration between the 4-week pre-treatment and the cumulative 8-week DEN/CCL_4_ exposure, which may have influenced observable protective effects.

## Discussion

The liver is one of the most vital solid organs in the human body, responsible for a wide array of essential metabolic, detoxifying, secretory, and regulatory functions that maintain physiological homeostasis. It serves as the principal site for the biotransformation and clearance of xenobiotics, including pharmaceutical compounds and toxic agents^26^. However, this central role also makes the liver susceptible to damage from environmental toxins and carcinogens.

HCC ranks as the fourth leading cause of cancer-related mortality worldwide, with limited therapeutic options available^27–29^; Alqurashy et al., 2025. Thus, there is a growing need to develop safer and more effective treatment strategies. Natural bioactives have emerged as promising candidates due to their broad pharmacological effects and generally favorable safety profiles^30–32^. Among these, BRB, a plant-derived isoquinoline alkaloid, has shown efficacy in treating various malignancies^18,33^. Despite this potential, BRB suffers from poor gastrointestinal absorption, low membrane permeability, and instability, which significantly reduce its clinical effectiveness^34^.

To address these limitations, nano-based drug delivery systems have been developed to enhance BRB’s bioavailability and therapeutic performance while minimizing systemic toxicity^35^. In the current study, we employed the same previously formulated and fully characterized batch of BRB-BSA NPs prepared by the desolvation method, as reported in Younis et al.,^17^ and Zaied et al.,^18^, ensuring identical formulation parameters, particle size, polydispersity, and zeta potential. This nanoformulation enhances the pharmacokinetic and pharmacodynamic properties of BRB. A combined DEN and CCl₄ regimen was used to induce a chemically induced chronic liver injury model rather than a full HCC model, consistent with the study objective. DEN generates reactive intermediates that cause DNA alkylation, oxidative stress, and early tumor-initiating events^36^. CCl₄, on the other hand, is a classic hepatotoxin that causes hepatocellular injury and fibrosis through free radical formation^37^.

In this context, BRB is traditionally used for its antimicrobial and antidiabetic effects. Although commercially available, its therapeutic utility is limited by strong plasma protein binding and poor bioavailability. Nanoparticle encapsulation is effective in addressing these challenges and improves solubility and systemic availability^17,18,38^.

The DEN/CCl₄ model displayed a pronounced oxidative imbalance, with elevated NO and XO levels, and reduced SOD activity. Notably, the vehicle/NP group also showed slightly improved redox markers. This likely reflects the intrinsic redox-buffering and reactive species-scavenging properties of albumin-based NPs, which may contribute to these modest improvements. Serum uric acid elevation observed in the DEN/CCl₄ group is suggestive of impaired renal function^39^, a feature seen in late-stage disease (Alqurashy et al.,^40^. The BRB-BSA NPs reversed these changes and improved antioxidant defense. Furthermore, uric acid reduction may relate to BRB’s nephroprotective effects^41^.

Autophagy, a cellular recycling process that removes damaged proteins and organelles, can either suppress tumor initiation or support tumor progression depending on the context. DEN/CCl₄ suppressed autophagy by upregulating PI3K and mTOR^42^. BRB-BSA NPs restored autophagic activity via PI3K/mTOR downregulation and LC3 upregulation, suggesting activation of autophagic cell death. Previous studies have demonstrated the ability of BRB to modulate autophagy^43^. In parallel, our docking analysis showed that BRB binds stably within the PI3K active site, supporting PI3K as a plausible molecular target, in a manner consistent with the observed in vivo decrease of PI3K/mTOR signaling. However, docking alone cannot establish direct target engagement. To contextualize these findings, it is important to note that unlike synthetic PI3K inhibitors that directly target the kinase domain, BRB-BSA NPs exert their effects through multimodal biological pathways, including modulation of oxidative stress, autophagy, and apoptosis. Although not intended to replace clinically approved PI3K inhibitors, BRB-BSA NPs provide several advantages, including natural origin, lower toxicity, improved bioavailability achieved through nanoparticle encapsulation, and broader hepatoprotective activity. Thus, BRB-BSA NPs should be viewed as a complementary therapeutic approach, one that influences PI3K signaling indirectly, consistent with both the docking predictions and the in vivo reduction in PI3K/mTOR activation.

Apoptosis was also impaired by DEN/CCl₄, as reflected by the imbalance between anti-apoptotic (Bcl-2) and pro-apoptotic (Bax) markers, favoring cell survival. BRB-BSA NPs effectively reversed this trend by reducing Bcl-2 expression and increasing Bax levels, thereby restoring apoptotic signaling and supporting their therapeutic potential^44^.

**Fig. 5:**
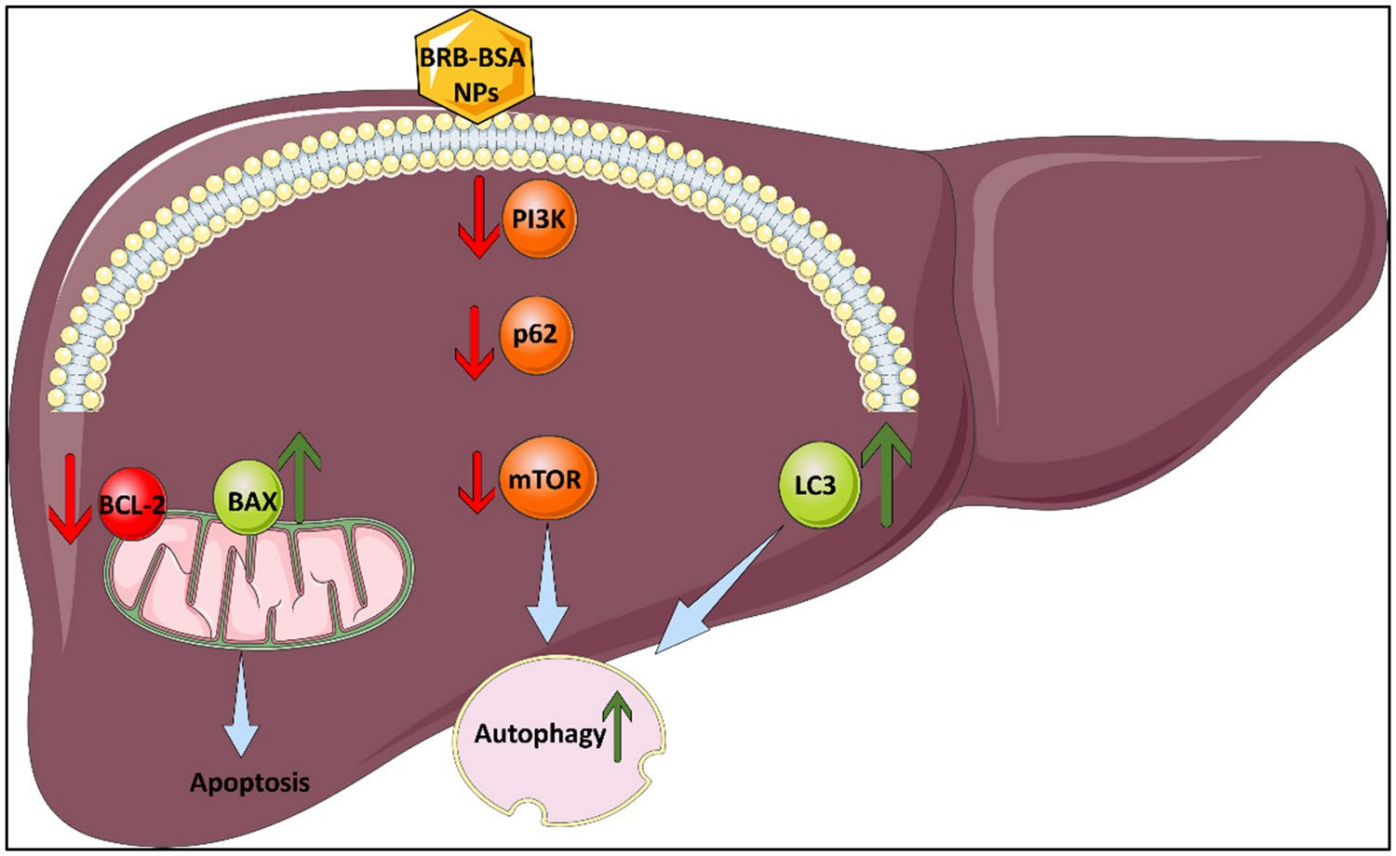
Schematic illustration of the modulatory effects of BRB-BSA NPs on PI3K/p62/SQSTM1/mTOR signaling, autophagy (LC3), and apoptotic markers (Bcl-2 and Bax).

The schematic illustration effectively summarizes the proposed hepatoprotective mechanism of BRB-BSA NPs through the modulation of key molecular pathways related to autophagy and apoptosis (Fig. 5). The image shows the liver as the target organ and depicts how BRB-BSA NPs, upon cellular internalization, downregulate PI3K, p62, and mTOR, key components in the PI3K/mTOR signaling axis known to inhibit autophagy. The suppression of these molecules leads to enhanced autophagic flux, as evidenced by the upregulation of LC3 and autophagic activity. Simultaneously, the diagram indicates a shift in the apoptotic balance by downregulating anti-apoptotic Bcl-2 and upregulating pro-apoptotic Bax, leading to apoptosis regulation. This dual action, enhancing autophagy while controlling apoptosis, highlights the therapeutic potential of BRB-BSA NPs in mitigating liver injury, particularly in conditions involving oxidative stress or toxin-induced damage.

Histopathological evaluation provided qualitative visual support for the biochemical and molecular findings, demonstrating tissue-level changes that aligned with reductions in oxidative damage and inflammation. Given that histology was conducted for corroborative purposes rather than as a primary quantitative endpoint, comparative interpretations (prophylactic versus therapeutic) are based primarily on statistically supported biochemical outcomes, with histology serving to reinforce the observed trends.

In conclusion, BRB-BSA NPs alleviated chronic liver injury induced by DEN/CCl₄ in male rats through modulation of oxidative stress, autophagy, and apoptosis pathways. However, these results should be interpreted with caution, as they represent preclinical data generated in a chemically induced rat model of chronic hepatic damage and cannot yet be directly extrapolated to human HCC.

Study limitations include the use of only male animals, and potential sex-specific differences were not assessed. Also, molecular docking provided predictive insights into PI3K interactions, but functional in vitro or ex vivo validation of direct PI3K inhibition by BRB was not performed. Although several key oxidative stress, autophagy, and apoptosis markers were analyzed, other relevant signaling cascades (e.g., NF-κB, or JNK pathways) were not investigated, limiting the mechanistic depth of the study. Furthermore, the duration of treatment was relatively short, and the long-term safety, pharmacokinetics, and biodistribution of BRB-BSA NPs were not evaluated. Finally, translation of these results to clinical settings requires further validation in human cell lines, additional animal models, and ultimately clinical trials.

Taken together, BRB-BSA NPs show promise as hepatoprotective agents, but further mechanistic and translational studies are required before clinical application can be considered.

## Data Availability

All data supporting the results of this study are included within the article. Additional raw datasets are available from the corresponding author upon reasonable request.

## Declarations

### Declaration of AI-assisted technologies in the writing process

During the revision of the original draft of this work the author(s) used ChatGPT AI in order to assist with copy editing for readability and style, and to ensure that the texts are free of errors in grammar, spelling, and punctuation. Afterthat, the author(s) reviewed and edited the content.
